# Protein tyrosine kinase Src suppresses hepatitis C virus particle release through regulation of Ndrg1

**DOI:** 10.1016/j.jbc.2025.111125

**Published:** 2025-12-30

**Authors:** Leihua Fu, Kenji Takeuchi, Kazuyasu Chihara, Weiying Feng, Kiyonao Sada

**Affiliations:** 1 Department of Genome Science and Microbiology, Faculty of Medical Sciences, University of Fukui, Fukui, Japan; 2 Department of Hematology, Shaoxing People’s Hospital, Shaoxing City, Zhejiang Province, People's Republic of China; 3 Organization for Life Science Advancement Programs, University of Fukui, Fukui, Japan

**Keywords:** hepatitis C virus (HCV), N-myc downstream regulated 1 (Ndrg1), non-receptor tyrosine kinase (nRTK), tyrosine kinase inhibitors, Src, tyrosine–protein kinase (tyrosine kinase)

## Abstract

Tyrosine kinases are known to regulate multiple stages of the hepatitis C virus (HCV) life cycle. We previously demonstrated that Abl kinase facilitates viral particle assembly; however, the roles of other tyrosine kinases remain largely undefined. In this study, we evaluated the antiviral potential of tyrosine kinase inhibitors (TKIs) and investigated the associated host regulatory mechanisms. Screening a panel of clinically approved TKIs in HCV-infected Huh-7.5 cells revealed that Bosutinib, a dual inhibitor of Abl and Src kinases, significantly reduced extracellular viral titers. Unexpectedly, CRISPR/Cas9-mediated knockout of Src kinase had no effect on viral replication, protein synthesis, or assembly but markedly enhanced the release of infectious particles. We further identified N-myc downstream regulated 1 (Ndrg1), a lipid metabolism regulator, as a downstream effector of Src. In Src-knockout cells, Ndrg1 expression was significantly downregulated at both the mRNA and the protein levels. Silencing Ndrg1 similarly promoted the release of infectious virions without affecting viral replication, indicating that the Src-Ndrg1 axis acts as a negative regulator of HCV egress. We further showed that Src kinase regulates Ndrg1 transcription *via* the Stat3-Hif1α signaling pathway. This previously unrecognized mechanism deepens our understanding of host–viral interactions and highlights a potential concern for patients with chronic HCV infection undergoing TKI-based therapies.

Hepatitis C virus (HCV), an enveloped positive-sense RNA virus of the Flaviviridae family, chronically infects more than 58 million people worldwide, with nearly 80% progressing to persistent infection and increased risk of liver cirrhosis and hepatocellular carcinoma (HCC) (1, 2). Upon viral entry and uncoating, its ∼9.6 kb genome is translated into a single polyprotein of over 3000 amino acids, which is cleaved by host and viral proteases into three structural proteins (core, E1, and E2) and seven nonstructural proteins (p7, NS2, NS3, NS4A, NS4B, NS5A, and NS5B) (3). The nonstructural proteins assemble on endoplasmic reticulum-derived membranes to form the RNA replication complex (4). This complex synthesizes positive-strand viral RNA, which is packaged with structural proteins to form nascent virions. The progeny virions bud into the endoplasmic reticulum lumen, pass through the Golgi apparatus, and are subsequently released from infected cells *via* the secretory pathway (3, 4).

The HCV life cycle is a complex process involving numerous host factors that play critical regulatory roles (5). Among these, the tyrosine kinase family, including both receptor tyrosine kinases (*e*.*g*., Egfr) and non-receptor tyrosine kinases (*e*.*g*., Abl and Src family members), has been implicated in key stages of viral propagation (6, 7, 8). Egfr has been shown to function as a co-factor that facilitates the activity of four critical HCV entry factors, including CD81, scavenger receptor class B type I, occluding, and claudin-1, during the viral entry stage (9, 10). We previously showed that either pharmacological inhibition or genetic knockout of Abl significantly suppresses HCV particle assembly (7, 8). Members of the Src family kinases (SFKs), including Hck, Lck, Lyn, and Fyn, have also been reported to interact with the viral nonstructural protein NS5A *via* their Src homology (SH) 3 domains (11), although their precise roles in the viral life cycle remain undefined. With the growing clinical use of tyrosine kinase inhibitors (TKIs), substantial therapeutic benefits have been achieved in many malignancies, leading to prolonged survival (12, 13). A prominent example is chronic myeloid leukemia, in which TKI treatment has led to a 10-year survival rate of approximately 85% (14). However, the potential impact of these drugs on patients co-infected with HCV remains unclear. Therefore, elucidating the functional roles of tyrosine kinases in HCV infection may offer important insights for therapeutic decisions in patients undergoing TKI treatment.

Moreover, although hepatitis C treatment has dramatically improved with the advent of direct-acting antivirals (15), which target viral proteins, new issues have arisen, including the emergence of resistant viruses and the persistence of refractory cases associated with liver cancer or underlying disease after viral elimination (16, 17). The possibility of future hepatitis C outbreaks as imported infectious diseases caused by novel viral genotypes cannot be ruled out. Compared to hepatitis B virus, HCV is more prone to developing resistance (18). Thus, there is an urgent need to address drug resistance that has accumulated during chronic infection and to develop therapeutic agents with novel mechanisms of action that are effective against a broad spectrum of HCV genotypes, including those associated with imported infections.

In this study, we systematically evaluated the effects of clinically approved TKIs on HCV propagation. Through CRISPR-Cas9-mediated genetic ablation and transcriptomic profiling, we further demonstrate that Src kinase functions as a negative regulator of HCV particle release by modulating N-myc downstream regulated 1 (Ndrg1), a lipid metabolism-associated factor (19). These findings reveal a previously unrecognized Src-Ndrg1 regulatory axis that modulates HCV egress, offering new mechanistic insights into how host factors govern the late stages of the HCV life cycle.

## Results

### Functional screening of tyrosine kinase inhibitors reveals distinct impacts on intra- and extracellular HCV titers

**Figure 1:**
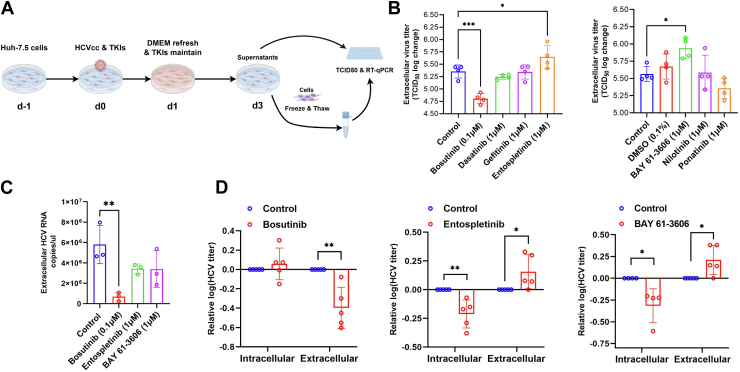
**Tyrosine kinase inhibitors differentially modulate intra- and extracellular HCV infectious particle production**. *A*, schematic overview of the experimental workflow. Huh-7.5 cells were seeded 1 day prior to infection (d-1), infected with HCVcc (MOI = 5) and treated with TKIs on day 0 (d0). After 24 h, the medium was refreshed and TKIs were re-administered (d1). After an additional 48 h of incubation (d3), culture supernatants and cell lysates were collected for TCID_50_ assays and RT-qPCR. *B*, Quantification of extracellular HCV infectivity following TKI treatment. Naïve Huh-7.5 cells were inoculated with supernatants collected 72 h post-infection from TKI-treated cultures. Infectious titers were determined by TCID_50_ assays using immunostaining for the HCV core protein. *C*, Extracellular HCV RNA was extracted and quantified by RT-qPCR. Viral RNA copy numbers were normalized to copies per microliter of supernatant. *D*, Comparison of intracellular and extracellular HCV titers following treatment with selected TKIs. Relative changes are shown for Bosutinib (*left*), Entospletinib (*middle*), and BAY 61-3606 (*right*) compared to untreated controls. Data are presented as mean ± SD from at least three independent biological replicates. Statistical significance was analyzed using One-way ANOVA followed by the Dunnett’s multiple comparisons test (*B*, *C*) or Mann-Whitney U test (*D*). ∗*p* < 0.05, ∗∗*p* < 0.01, ∗∗∗*p* < 0.001.

We evaluated the impact of a series of clinically approved TKIs on the production of infectious HCV particles. Initially, to determine the optimal concentrations of TKIs for subsequent assays, we first performed cell proliferation inhibition assay (Fig. S1). Based on these results, selected TKIs were applied to HCV-infected cells to assess their effects on viral infectivity. Huh-7.5 cells were seeded in 24-well plates 1 day prior to infection and subsequently infected with cell culture-derived HCV (HCVcc) at a multiplicity of infection (MOI) of 5 and TKIs at their optimized concentrations. After 24 h, the medium was replaced with fresh medium containing the same concentrations of TKIs, and cells were further incubated for an additional 48 h (Fig. 1*A*). As shown in Fig. 1*B*, treatment with Bosutinib or either of the two Syk kinase inhibitors, BAY 61-3606 and Entospletinib, significantly modulated extracellular viral titers (*p* < 0.05). Specifically, Bosutinib markedly suppressed extracellular HCV titers, whereas BAY 61-3606 and Entospletinib markedly increased the viral titers. In contrast, other TKIs, including Dasatinib and Ponatinib, did not exert measurable effects on extracellular infectivity.

We next measured extracellular viral RNA copies and found that Bosutinib treatment markedly reduced extracellular RNA levels by approximately 88%, whereas the two Syk inhibitors had no significant effect (Fig. 1*C*). We also quantified intracellular viral titers to further assess the impact of these treatments. As shown in Fig. 1*D*, treatment with BAY 61-3606 and Entospletinib resulted in a significant reduction in intracellular viral titers, whereas Bosutinib exerted negligible effects on intracellular HCV levels.

### Tyrosine kinase inhibitors treatment does not affect HCV entry or viral replication

The dual Abl/Src kinase inhibitor Bosutinib, along with two Syk kinase inhibitors, BAY 61-3606 and Entospletinib, were found to modulate the production of infectious HCV particles. To investigate whether these compounds affect viral infection efficiency or replication, we performed immunofluorescence staining, RT-qPCR, and immunoblotting analyses.

**Figure 2:**
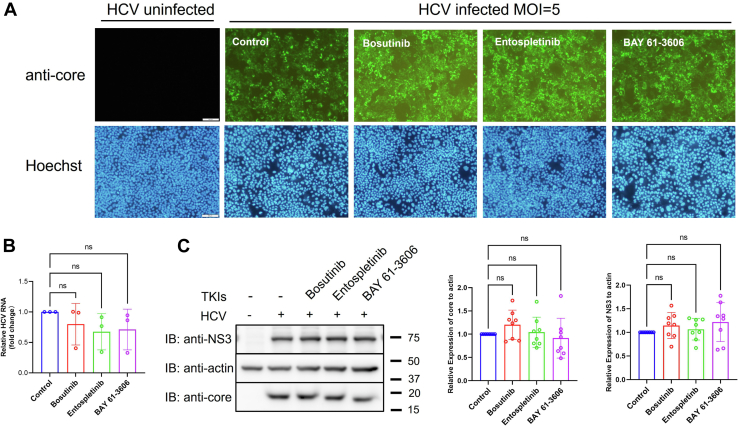
**Effect of tyrosine kinase inhibitors on HCV infection efficiency and viral protein expression**. *A*, Huh-7.5 cells were infected with HCVcc (MOI = 5) and treated with Bosutinib, Entospletinib, or BAY 61-3606 following the protocol described in Fig. 1*A*. At 72 h post-infection, cells were fixed and stained with an anti-core monoclonal antibody (*top panels*) and Hoechst to visualize nuclei (*bottom panels*). Scale bar, 100 μm. *B*, quantification of intracellular HCV RNA levels treated with the indicated inhibitors. Total RNA was extracted and analyzed by RT-qPCR. Data represent the relative expression normalized to untreated controls. *C*, immunoblot analysis of HCV NS3 and core protein expression. Detergent-soluble lysates were prepared from infected cells treated with the indicated inhibitors, separated by SDS-PAGE, and probed with anti-NS3, anti-core, and anti-actin antibodies. Densitometric quantification of NS3 and core protein levels relative to actin is shown in the *right panels*. Data are presented as mean ± SD from at least three independent biological replicates. Statistical significance was analyzed using Welch’s *t* test. ns, not significant. IB, immunoblot.

Immunostaining of HCV-infected cells demonstrated comparable levels of core protein expression between TKI-treated and untreated groups (Fig. 2*A*), suggesting that TKI treatment did not impair viral entry or initial infection. Consistent with this observation, intracellular HCV RNA levels, as quantified by RT-qPCR, were not significantly altered following TKIs treatment (Fig. 2*B*). Furthermore, immunoblot analysis revealed no notable differences in the expression levels of HCV NS3 or core proteins among the various treatment conditions (Fig. 2*C*). Collectively, these data indicate that the tested TKIs do not interfere with viral entry, RNA replication, or protein synthesis, but may exert their effects at post-replication stages of the viral life cycle.

In conjunction with the viral titration results and extracellular viral RNA copies measurements, we speculate that the two Syk inhibitors, BAY 61-3606 and Entospletinib, may modulate virion maturation and thereby increase the specific infectivity of released particles, although virion maturation was not directly assessed in this study. The observed reduction in intracellular viral titers may therefore reflect a secondary effect of increased virion release, rather than a defect in viral assembly. Since the *syk* gene has been reported to be epigenetically silenced by promoter hypermethylation in several HCC cell lines, including Huh-7 cells (20), we were unable to detect Syk protein expression in our system (data not shown). Thus, it is likely that the effects observed upon treatment with these inhibitors were mediated through off-target mechanisms rather than direct inhibition of Syk kinase.

We then focused on Bosutinib, a dual inhibitor of Src and Abl kinases, which significantly suppressed extracellular HCV titers and viral RNA copies without affecting intracellular levels, suggesting that it likely blocks the virus at the stage of release.

### Comprehensive analysis of transcriptomic alterations reveals candidate genes and pathways regulating HCV release

**Figure 3:**
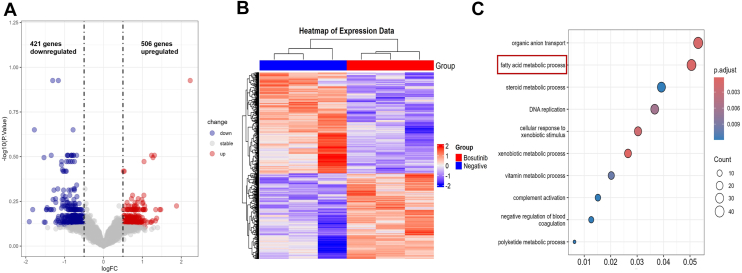
**Transcriptomic profiling of HCV-infected hepatocytes treated with Bosutinib**. *A*, Volcano plot showing DEGs between Bosutinib-treated and untreated (negative control) HCV-infected Huh-7.5 cells. A total of 506 genes were upregulated (*red*) and 421 genes were downregulated (*blue*) based on the thresholds of |log_2_FC| > 0.5 and *p* < 0.05 (n = 3). *B*, heatmap depicting hierarchical clustering of DEGs between the two groups. Each column represents a sample, and each row represents a gene. *Red* indicates high expression, and *blue* indicates low expression. *C*, gene ontology enrichment analysis of DEGs. The top 10 significantly enriched biological processes are shown, including fatty acid metabolic process, steroid metabolic process, and DNA replication. Dot size corresponds to gene count, and color indicates adjusted *p*-value.

Our data demonstrated that Bosutinib significantly reduced extracellular HCV particles (Fig. 1*C*). We therefore thought of elucidating the molecular mechanisms underlying this regulatory effect. To this end, transcriptomic profiling was performed using microarray analysis in HCV-infected hepatocytes treated with or without Bosutinib. A total of 927 differentially expressed genes (DEGs) were identified, including 506 upregulated and 421 downregulated genes (Fig. 3*A* and *B*, Table S1).

Gene ontology enrichment analysis revealed that Bosutinib treatment significantly altered several biological processes, some of which are potentially implicated in viral release. Notably, genes involved in fatty acid metabolic processes, steroid metabolic processes, and xenobiotic metabolism were among the most significantly enriched pathways (Fig. 3*C*).

### Src kinase functions as a negative regulator of HCV particle release

**Figure 4:**
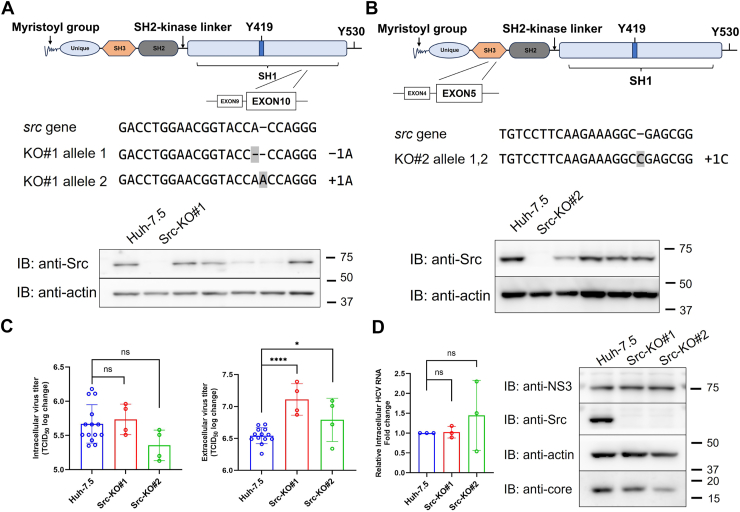
**Generation of Src-KO cells and evaluation of their effect on HCV production**. *A–B*, structure of human Src kinase and its variants generated *via* genome editing. CRISPR/Cas9-mediated gene editing induced indel mutations targeting exon 10 in Src-KO#1 cells (*A*) and exon 5 in Src-KO#2 cells (*B*). The nucleotide sequences of wild-type Src and mutated alleles are shown below the schematic diagrams. *Gray* boxes indicate insertion or deletion sites. Immunoblotting analysis confirmed the loss of Src protein expression in the knockout clones. *C*, TCID_50_ assay to evaluate intra- and extracellular HCV production. Cells were infected with HCV, and both cell lysates and culture supernatants were collected at 72 h post-infection. *Left*: intracellular virus titers. *Right*: extracellular virus titers. *D*, RT-qPCR analysis showed no significant differences in HCV RNA levels among Huh-7.5, Src-KO#1, and Src-KO#2 cells. Immunoblot analysis further confirmed that NS3 protein expression remained unchanged in both wild-type and Src-KO cells, while core protein expression was slightly reduced in Src-KO cells (the same membrane was cut into four pieces). Data are presented as mean ± SD from at least three independent biological replicates. Statistical significance was analyzed using One-way ANOVA followed by the Dunnett’s multiple comparisons test (*C*) or Welch’s *t* test (*D*). ∗*p* < 0.05, ∗∗∗∗*p* < 0.0001, ns, not significant.

Bosutinib is a dual inhibitor of both Abl and Src kinases. This raised the question of whether the observed effect results from the combined inhibition of both targets. Our previous studies demonstrated that pharmacological inhibition or knockout of Abl kinase suppresses HCV assembly (7, 8). To investigate the specific role of Src kinase in the HCV life cycle, we established two Src-knockout (Src-KO) Huh-7.5 cell lines using CRISPR/Cas9-mediated genome editing, with single-guide RNAs targeting distinct exons (Fig. 4*A* and *B*). Src-KO#1 cells harbored two distinct indels in exon 10, while Src-KO#2 carried biallelic +1C insertions in exon 5 (Fig. 4, *A* and *B*, middle panel). Complete abrogation of Src protein expression was confirmed by immunoblotting in both knockout clones (Fig. 4*A* and *B*, bottom panel).

We first examined extracellular HCV titers in Src-KO cells. Unexpectedly, extracellular viral titers were significantly increased in both Src-KO clones compared to parental cells, with approximately 3.7-fold and 1.8-fold increases observed in Src-KO#1 and Src-KO#2, respectively (Fig. 4*C*, right panel; *p* < 0.0001 for KO#1, *p* < 0.05 for KO#2). In contrast, intracellular viral titers remained comparable (Fig. 4*C*, left panel), indicating enhanced release of infectious virions. We then assessed both intra- and extracellular HCV RNA levels. Consistently, intracellular viral RNA levels were similar between knockout and parental cells (Fig. 4*D*, left panel), whereas extracellular HCV RNA levels were markedly elevated in Src-KO cells, showing approximately 3.4-fold and 1.9-fold increases in Src-KO#1 and Src-KO#2, respectively (Fig. S2). Immunoblot analysis further confirmed that expression of the viral nonstructural protein NS3 was unchanged in both wild-type and Src-KO cells, suggesting that Src deletion does not affect viral replication or protein synthesis. We found that core protein expression was slightly reduced in Src-KO cells (Fig. 4*D*, right panel), likely reflecting the increased release of virions from infected cells.

Together, these findings support a model in which Src kinase restricts the late stages of the HCV life cycle, particularly virion release, suggesting that the antiviral effect of Bosutinib does not fully result from the combined inhibition of both Src and Abl kinases. The respective roles of Bosutinib, Abl kinase, and Src kinase in distinct stages of the viral life cycle are summarized in Table 1.

**Table 1:** Summary of effects on HCV titers under pharmacological inhibition (Bosutinib) or gene knockout conditions

HCV titer	Bosutinib	Genome-edited cells	
	Abl/Src kinase inhibited	Abl-KO	Src-KO
Intracellular	ns	Reduce	ns
Extracellular	Reduce	Reduce	Enhance

“ns” indicates no significant difference compared to the control.

### Ndrg1 negatively regulates HCV particle release

**Figure 5:**
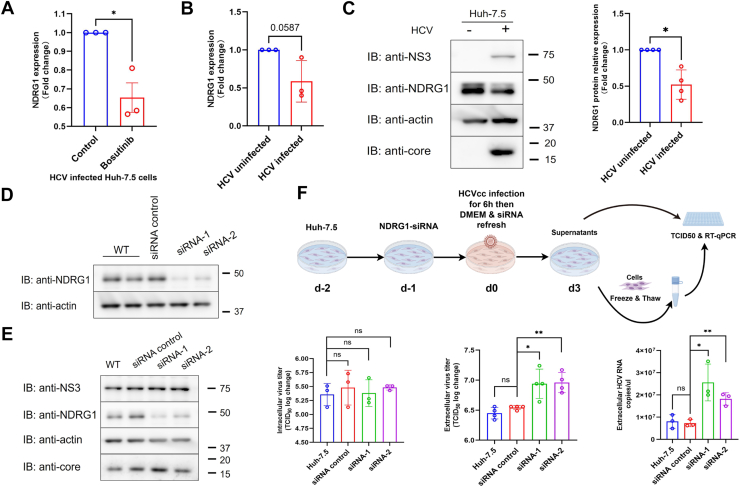
**Ndrg1 negatively regulates HCV virion release without affecting intracellular viral production**. *A*, RT-qPCR analysis of Ndrg1 mRNA expression in HCV-infected Huh-7.5 cells with or without Bosutinib treatment. Bosutinib significantly reduced Ndrg1 expression compared to the control group. *B*, a trend toward downregulation of Ndrg1 mRNA expression was observed in HCV-infected Huh-7.5 cells (*p* = 0.0587). *C*, Immunoblot analysis confirming reduced Ndrg1 protein expression in HCV-infected cells. *D*, efficient knockdown of Ndrg1 in Huh-7.5 cells was validated by immunoblotting using two independent siRNAs. *E*, immunoblot analysis of HCV-infected cells treated with control or Ndrg1 siRNAs showed that knockdown of Ndrg1 did not alter the expression levels of NS3 and core proteins, indicating no effect on intracellular viral protein production. *F*, TCID_50_ assay and RT-qPCR analysis to assess intra- and extracellular HCV production in Ndrg1-knockdown cells. Schematic of the experimental workflow (*top*). Intracellular viral titers remained unchanged, while extracellular viral titers and RNA copies were significantly increased in Ndrg1-knockdown cells compared to wild-type and siRNA controls (*bottom panel*). Data are presented as mean ± SD from at least three independent biological replicates. Statistical significance was analyzed using Welch’s *t* test (*A*, *B*, *C*) or One-way ANOVA followed by the Dunnett’s multiple comparisons test (*F*). ∗*p* < 0.05, ∗∗*p* < 0.01, ns, not significant.

Our data clearly demonstrated that Src kinase restricts the release stage of the HCV life cycle without affecting viral replication or infectious particle assembly. Therefore, we speculated that certain genes involved in viral release might be regulated by Src kinase. We noted that our transcriptomic profiling revealed significant enrichment of several pathways closely associated with the viral life cycle (Fig. 3*C*), including the fatty acid metabolic process (21). To identify genes that may be involved in viral egress, we further examined the DEGs identified in microarray analysis. Among these, we found that Ndrg1, which has been reported to negatively regulate the incorporation of fatty acids into neutral lipids and lipid droplet formation (19), was significantly downregulated in our microarray analysis (log_2_ fold change (FC) = −0.61, *p* = 0.028; Table S1). This result was further validated by RT-qPCR (Fig. 5*A*), confirming that Bosutinib suppresses Ndrg1 expression at the transcriptional level.

In addition, we observed that both mRNA and protein levels of Ndrg1 were decreased following HCV infection, suggesting its potential involvement in a specific stage of the viral life cycle (Fig. 5, *B* and *C*). To investigate this, we performed siRNA-mediated knockdown of Ndrg1 using two independent sequences. Immunoblotting analysis confirmed effective knockdown of Ndrg1, with knockdown efficiencies of approximately 82% for siRNA-1 and 65% for siRNA-2 (Fig. 5*D*). We then examined the levels of HCV NS3 and core proteins under Ndrg1 knockdown conditions. Neither NS3 nor core protein expression was significantly affected (Fig. 5*E*), indicating that Ndrg1 does not affect viral protein synthesis.

Subsequent viral titration assays revealed that silencing Ndrg1 significantly enhanced extracellular viral titers, increasing by 2.4-fold with siRNA-1 and 2.5-fold with siRNA-2, while intracellular titers remained unchanged (Fig. 5*F*, left and middle panels). Consistently, measurement of extracellular viral RNA copies revealed a marked increase upon Ndrg1 knockdown, increasing by 3.5-fold with siRNA-1 and 2.5-fold with siRNA-2 (Fig. 5*F*, right panel). These findings indicate that Ndrg1 acts as a negative regulator of HCV particle release, rather than influencing viral replication or assembly. This observation is consistent with the finding in Src-KO cells. In addition, treatment with Bosutinib, a Src kinase inhibitor, led to a marked downregulation of Ndrg1 expression. These findings prompted us to explore whether Ndrg1 functions as a downstream effector of Src signaling.

### Src kinase regulates HCV particle release *via* downregulation of Ndrg1

**Figure 6:**
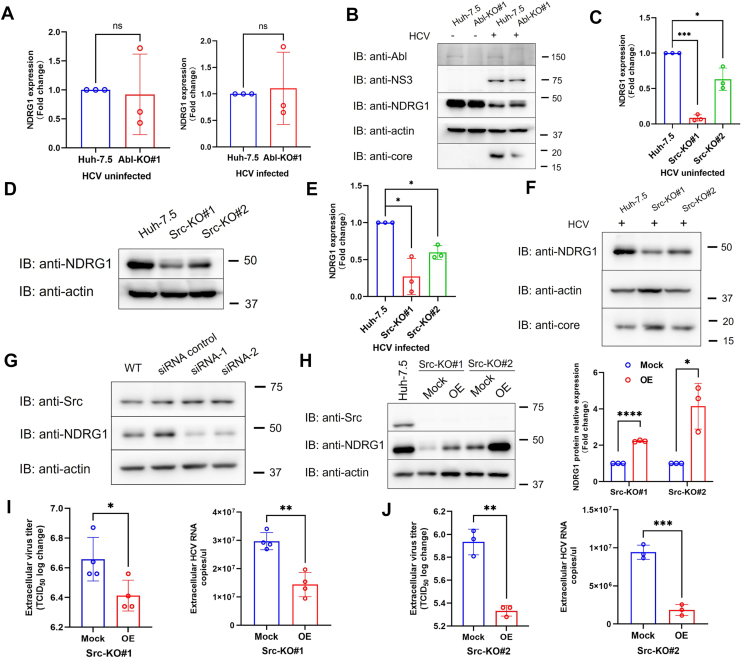
**Src kinase regulates Ndrg1 expression and HCV particle release**. *A*, RT-qPCR analysis of Ndrg1 mRNA expression in Abl-KO#1 Huh-7.5 cells. No significant differences in Ndrg1 transcript levels were observed between Abl-KO#1 and wild-type cells under either uninfected (*left*) or HCV-infected (*right*) conditions. *B*, immunoblot analysis confirmed that Ndrg1 protein expression remained unchanged in Abl-KO#1 cells compared to normal Huh-7.5 cells. *C*, RT-qPCR analysis of uninfected Src-KO#1 and Src-KO#2 clones showed significantly reduced Ndrg1 mRNA expression relative to Huh-7.5 cells. *D*, immunoblotting confirmed a marked reduction in Ndrg1 protein levels in uninfected Src-KO clones. *E*, RT-qPCR analysis of HCV-infected cells revealed significantly decreased Ndrg1 mRNA expression in Src-KO#1 and Src-KO#2 clones. *F*, immunoblot analysis further demonstrated suppression of Ndrg1 protein in Src-KO clones following HCV infection. *G*, immunoblot analysis of cells transfected with Ndrg1-targeting siRNAs confirmed that Ndrg1 knockdown did not affect Src protein levels. *H*, immunoblot analysis showing Ndrg1 expression in Src-KO#1 and Src-KO#2 cells following transient transfection with an Ndrg1-overexpressing plasmid (OE) or an empty vector (Mock). Quantification of Ndrg1 protein levels showed that relative Ndrg1 expression increased by 2.3-fold in Src-KO#1 and by 4.1-fold in Src-KO#2 compared with the corresponding Mock controls. *I* and *J*. Extracellular HCV RNA copies and viral titers in Src-KO#1 (*I*) and Src-KO#2 (*J*) cells, with or without Ndrg1 overexpression. Data represent the mean ± SD from three or more independent biological replicates. Statistical significance was analyzed using Welch’s *t* test (*A*, *C*, *E*, *H*) and two-tailed unpaired Student’s *t* test (*I* and *J*). ∗*p* < 0.05, ∗∗*p* < 0.01, ∗∗∗*p* < 0.001, ∗∗∗∗*p* < 0.0001, ns, not significant.

Although Bosutinib was shown to suppress Ndrg1 expression (Fig. 5*A*), it remained unclear whether this effect was specifically mediated through Src kinase inhibition, as the contribution of Abl kinase inhibition could not be excluded. To clarify this, we first assessed Ndrg1 mRNA and protein expression in two Abl-knockout (Abl-KO) cell lines (7). The results demonstrated that genetic ablation of Abl kinase did not alter Ndrg1 expression at either the transcript or protein level (Fig. 6, *A* and *B*, Fig. S3), suggesting that Abl is not involved in the regulation of Ndrg1 under these conditions.

To further determine whether Ndrg1 suppression is linked to Src inhibition, we analyzed Ndrg1 expression in Src-KO cells. We found that both mRNA and protein levels of Ndrg1 were markedly reduced in Src-KO cells under both HCV-infected and uninfected conditions (Fig. 6, *C*–*F*), indicating that Src acts as an upstream regulator of Ndrg1. Notably, this regulatory relationship appeared to be independent of HCV infection. We further confirmed that knockdown of Ndrg1 did not alter total Src protein levels (Fig. 6*G*), supporting the notion that Ndrg1 functions downstream of Src signaling.

We next performed Ndrg1 overexpression experiments in Src-KO clones. Compared with the mock group, Ndrg1 expression increased by 2.3-fold in Src-KO#1 and 4.1-fold in Src-KO#2 (Fig. 6*H*), possibly reflecting clonal variation. We then assessed extracellular viral titers and RNA copies. In both clones, Ndrg1 overexpression markedly decreased viral titers and RNA copies (Fig. 6, *I* and *J*), indicating that restoring Ndrg1 suppresses the enhanced viral release observed in Src-KO cells.

Collectively, these findings support a model in which inhibition of Src kinase leads to downregulation of Ndrg1, thereby facilitating HCV particle release.

### Src kinase regulates Ndrg1 transcription *via* the Stat3-Hif1α signaling pathway

**Figure 7:**
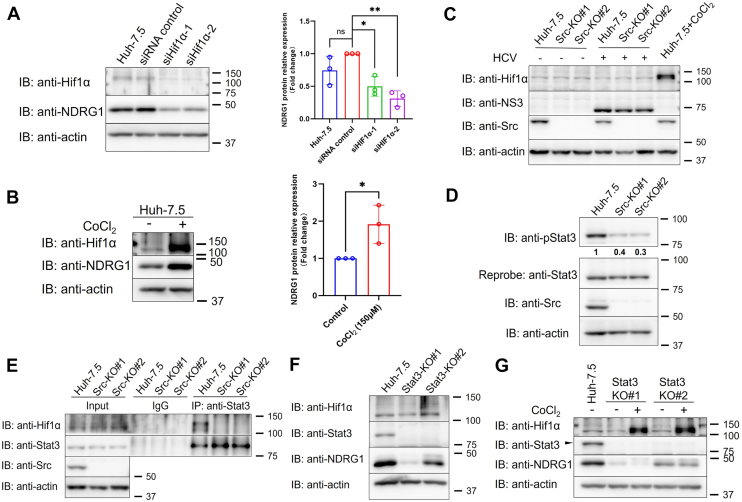
**Src kinase regulates Ndrg1 transcription via the Stat3-Hif1α signaling pathway**. *A*, silencing Hif1α decreases Ndrg1 expression. *B*, stabilization of Hif1α by CoCl_2_ upregulates Ndrg1 expression. Huh-7.5 cells were treated with CoCl_2_ (150 μM, 24 h). Right: quantification of Ndrg1 protein levels relative to control. *C*, Hif1α expression is not reduced in Src-KO cells. Huh-7.5, Src-KO#1, and Src-KO#2 cells were either infected or left uninfected with HCV (MOI = 5) for 72 h, followed by immunoblot analysis. Hif1α levels remained comparable across all cell lines. CoCl_2_ treatment (150 μM) served as a positive control for Hif1α stabilization. *D*, Src knockout reduces Stat3 Tyr705 phosphorylation. Densitometric ratios normalized to Huh-7.5 control are indicated below each blot. *E*. Loss of Src kinase disrupts the interaction between Hif1α and Stat3. The Stat3-Hif1α interaction was markedly reduced in both Src-KO clones. *F*. Ndrg1 expression is decreased in Stat3-KO cells. Loss of Stat3 significantly reduced Ndrg1 levels without altering Hif1α abundance. *G*, Stat3 is required for CoCl_2_-induced upregulation of Ndrg1. Stat3-KO#1, and Stat3-KO#2 cells were treated with or without CoCl_2_ (150 μM, 24 h). CoCl_2_-mediated induction of Ndrg1 was abolished in Stat3-KO cells. Data represent the mean ± SD of three independent biological replicates. Statistical significance was analyzed using Welch’s *t* test. ∗*p* < 0.05, ns, not significant.

Our data indicate that Src kinase regulates Ndrg1 at the transcriptional rather than the post-translational level (Fig. 6, *C*–*F*). Ndrg1 has been reported to be transcriptionally regulated by several upstream factors, including N-myc and hypoxia inducible factor 1 subunit alpha (Hif1α) (22). We initially examined N-myc mRNA levels in Src-KO cells and found that Src loss did not affect N-myc expression (Fig. S4). We therefore turned our attention to Hif1α. Hif1α, a hypoxia-inducible transcription factor, positively regulates Ndrg1 expression, and its expression has been reported to decrease following Src kinase inhibition (23). It has been shown that Hif1α is expressed under normoxic conditions in Huh-7 cells (24). Consistently, we detected Hif1α expression in Huh-7.5 cells, and silencing Hif1α markedly reduced Ndrg1 expression (Fig. 7*A*), whereas treatment with CoCl_2_, a hypoxia-mimetic agent that stabilizes Hif1α (25), significantly upregulated Ndrg1 expression (Fig. 7*B*), indicating that Ndrg1 is transcriptionally regulated by Hif1α.

Given these observations and previous reports suggesting that Src inhibition reduces Hif1α levels (23), we next assessed its expression in Src-KO cells and, unexpectedly, observed no significant decrease (Fig. 7*C*). This finding suggests that the reduction in Ndrg1 expression is unlikely due to changes in Hif1α abundance itself, but may instead result from impaired transcriptional activity of Hif1α. Previous studies have demonstrated that phosphorylation of signal transducer and activator of transcription 3 (Stat3) at Tyr705 (pStat3) is critical for optimal Hif1α signaling, as pStat3 cooperates with Hif1α to activate its target genes (26, 27, 28, 29, 30), including Ndrg1 (29). Since Src kinase functions as an upstream kinase that contributes to Stat3 Tyr705 phosphorylation (31), we hypothesized that the loss of Src kinase reduces Stat3 phosphorylation, thereby diminishing Stat3-Hif1α interaction and consequently lowering Ndrg1 expression. To validate this hypothesis, we examined Stat3 phosphorylation in Src-KO cells and found that pStat3 levels were decreased by approximately 60% and 70% in Src-KO#1 and Src-KO#2 cells, respectively (Fig. 7*D*). Co-immunoprecipitation analysis further confirmed that the interaction between Stat3 and Hif1α was substantially reduced in Src-KO cells (Fig. 7*E*). We next analyzed Ndrg1 expression in Stat3-KO cells (32). Consistent with the requirement of Stat3 for Hif1α transcriptional activity, Ndrg1 levels were significantly decreased in Stat3-KO cells, whereas Hif1α expression remained unaffected (Fig. 7*F*). Moreover, we found that the CoCl_2_-induced upregulation of Ndrg1 was abolished in Stat3-KO cells (Fig. 7*G*). These findings suggest that Src kinase-mediated phosphorylation of Stat3 facilitates the formation of a transcriptionally active Stat3-Hif1α complex, which cooperatively regulates Ndrg1 expression. Loss of Src kinase disrupts this signaling cascade, leading to reduced Ndrg1 transcription.

## Discussion

In this study, we evaluated the impact of several clinically used TKIs on the HCV life cycle. Among them, Bosutinib, a dual inhibitor of Abl and Src kinases, was found to suppress the production of infectious HCV particles (Fig. 1*B*). In contrast, treatment with two Syk kinase inhibitors resulted in increased release of infectious particles (Fig. 1*B*). However, this effect is unlikely to be directly mediated by Syk, as our microarray analysis revealed very low levels of *syk* mRNA expression (3.05 ± 0.47; mean ± SD, log_2_ scale), and Syk protein was undetectable under our experimental conditions. Notably, the *syk* gene was reported to be epigenetically silenced *via* promoter hypermethylation in several HCC cell lines, including Huh-7 cells (20), consistent with our observations.

Our further investigation revealed that the antiviral effect of Bosutinib on HCV is not entirely attributable to the combined inhibition of both Abl and Src kinases (Table 1). Specifically, Abl kinase has been shown to interact with the nonstructural protein NS5A, and knockout of Abl kinase leads to the suppression of HCV particle assembly, as demonstrated in our previous studies (7, 8). In the present work, we found that knockout of Src kinase did not affect viral entry, replication, or intracellular production of infectious particles (Fig. 4*C* left panel, Fig. 4*D*). Unexpectedly, however, it significantly enhanced the release of infectious HCV particles (Fig. 4*C* right panel, Fig. S2). These results suggest that the overall reduction in extracellular viral titers observed with Bosutinib treatment may be primarily driven by Abl inhibition. Since inhibition of Abl prevents the formation of fully assembled virions, the enhanced release induced by Src inhibition cannot compensate for the loss of particle integrity, resulting in a net decrease in extracellular infectivity. However, this mechanism does not fully explain the unchanged levels of intracellular infectious particles observed following Bosutinib treatment. Kinome-wide profiling revealed that Bosutinib targets a broad spectrum of tyrosine and serine/threonine kinases beyond Abl and Src, including members of the serine/threonine kinase 24, calcium/calmodulin dependent protein kinase II gamma, and tec protein tyrosine kinase families (33). This polypharmacology raises the possibility that off-target inhibition of additional host kinases may interfere with viral particle assembly or trafficking, thereby partially compensating for the effect of Abl inhibition and helping to preserve intracellular infectivity despite Bosutinib treatment.

Several members of the Src kinase family have been reported to interact with the HCV NS5A protein (11). A previous study showed that Src kinase directly interacts with the viral NS5B protein *via* its SH3 domain and with NS5A *via* its SH2 domain, forming a complex that is recognized as a prerequisite for viral replication (34). Knockdown of Src reduces viral replication and protein synthesis, and this effect cannot be rescued by other SFKs (34). However, in our Src-KO model, we clearly observed that neither viral replication nor protein synthesis was affected (Fig. 4*D*). One possible explanation for this discrepancy is that, in the knockdown model, residual Src protein remains present. These remaining proteins may still occupy key viral binding sites, such as the tyrosine residue at position 93 of NS5A (35), thereby preventing compensatory binding by other SFKs. However, the residual protein is likely insufficient to form a Src-NS5A-NS5B complex required for efficient replication and translation. In contrast, our knockout model resulted in the complete loss of Src kinase, which may have allowed other kinases with similar affinity to bind NS5A and functionally compensate for its absence. Other SFKs, such as Fyn, have also been shown to interact with NS5A *via* their SH2 domains in a phosphorylation-dependent manner (36), supporting the possibility of functional compensation. Such compensatory recruitment of alternative host factors likely reflects a viral adaptation mechanism to maintain its life cycle in the face of essential host factor loss. It would be of interest to identify which specific kinase(s) can substitute for Src function in this context.

While the loss of Src kinase does not impair viral replication or protein synthesis, it exerts a distinct effect on the late phase of the HCV life cycle. Our study showed that the absence of Src kinase significantly enhanced the release of infectious HCV particles (Fig. 4*C*, right panel). Src kinase is involved in the regulation of multiple genes and signaling pathways within cells (37), suggesting that its role in promoting viral particle release may be mediated through the modulation of specific signaling cascades or gene expression. Li Q *et al*. previously reported that knockdown of Ndrg1 resulted in a marked increase in the release of infectious HCV particles (38). Consistent with these findings, we observed that knockdown of Ndrg1 also promoted the release of infectious virions without affecting viral replication (Fig. 5, *E* and *F*). Moreover, loss of Src kinase led to a marked reduction in Ndrg1 expression, and this regulation occurred independent of HCV infection (Fig. 6, *C*–*F*). Restoration of Ndrg1 expression suppressed the enhanced viral release in Src-KO cells (Fig. 6, *H*–*J*). These observations indicate that the increased viral release observed in Src-KO cells is attributable to the downregulation of Ndrg1. Ndrg1 has been implicated in the regulation of lipid metabolism (19) and vesicular trafficking (39), processes that are essential for the assembly and release of enveloped viruses. It is therefore plausible that reduced Ndrg1 expression facilitates HCV particle release by altering lipid-associated trafficking pathways or membrane dynamics. Nevertheless, the precise molecular mechanisms by which Ndrg1 regulates virion egress remain to be elucidated.

Our findings suggest that Src regulates Ndrg1 expression at the transcriptional rather than the post-transcriptional level (Fig. 6, *C*–*F*), consistent with reports that Ndrg1 lacks a canonical Src-binding motif (39, 40). We further found that Src kinase regulates Ndrg1 transcription through the Stat3-Hif1α signaling pathway. Hif1α is an upstream activator of Ndrg1 (22), and we confirmed this regulation by either stabilizing Hif1α expression under hypoxia-mimetic conditions with CoCl_2_ treatment or silencing Hif1α using siRNA, both of which led to corresponding changes in Ndrg1 expression (Fig. 7, *A* and *B*). However, Src does not regulate Ndrg1 expression by altering Hif1α abundance (Fig. 7*C*), but rather by modulating its cooperation with Stat3 (Fig. 7*E*). Tyr705-phosphorylated Stat3 physically interacts with the bHLH and PAS domains of Hif1α (28) and cooperatively activates Hif1α target genes, including Ndrg1 (29). Consistently, our results showed that Stat3-KO abolished the CoCl_2_-induced upregulation of Ndrg1, supporting the role of Stat3 as a cooperative activator of Hif1α. In Src-KO cells, we observed a marked decrease in Stat3 phosphorylation (Fig. 7*D*), which impaired its interaction with Hif1α and resulted in decreased Ndrg1 transcription. Furthermore, our co-immunoprecipitation analysis showed that the interaction between Hif1α and Stat3 was nearly undetectable in Src-KO cells (Fig. 7*E*), indicating that Src kinase is required for the functional cooperation between Hif1α and Stat3. These findings suggest that Src not only regulates Stat3 phosphorylation but may also facilitate the formation or stabilization of the Hif1α transcriptional complex by modulating essential cofactors such as CBP/p300 and ARNT (Hif1b) (23), thereby ensuring optimal transcriptional activation of Ndrg1 and other Hif1α target genes.

There are only a few studies that have reported the involvement of tyrosine kinases in the life cycle of RNA viruses, such as tyrosine phosphorylation of the Ebola virus protein VP40 and the HCV nonstructural protein NS5A (7, 8, 41), our findings reveal a previously unrecognized host–viral regulatory axis in which Src kinase suppresses HCV particle release through modulation of Ndrg1. This expands the understanding of host control over the late stages of the HCV life cycle and highlights the therapeutic potential of targeting host kinases to disrupt viral propagation. Notably, current treatment strategies for HCC employ receptor-type TKIs (*e*.*g*., Vegf, Pdgf, Ret, and Flt-3) aimed at suppressing tumor growth and angiogenesis, and are increasingly combined with immune checkpoint inhibitors (42, 43). Although this study primarily focuses on non-receptor protein tyrosine kinases involved in the viral life cycle, our findings have potential for further development as therapeutic strategies targeting both antiviral therapy and inhibition of hepatocarcinogenesis. Moreover, given the widespread clinical application of TKIs, these results highlight the need to evaluate kinase-virus interactions in patients with concurrent HCV infection.

## Experimental procedures

### Tyrosine kinase inhibitors and antibodies

Second-generation Bcr-Abl TKIs, including Nilotinib (Selleck, S5205), Bosutinib (Selleck, S1014), and Dasatinib (Funakoshi, 11,498), as well as the third-generation TKI Ponatinib (Selleck, S1490), were used in this study. In addition, two Syk kinase inhibitors, Entospletinib (Selleck, S7523) and BAY 61-3606 (Selleck, S7006), and the Egfr inhibitor Gefitinib (Funakoshi, CS-0124) were also included in the analysis. All inhibitors were dissolved in DMSO and stored at −20 °C until use. CoCl_2_ (Fujifilm, 7646–79–9) was dissolved in ddH_2_O and store at −20 °C until use. Anti-HCV core monoclonal antibody (Abcam, catalog number: ab2740, lot number: 1076091–2), anti-NS3 polyclonal antibody (GeneTex, catalog number: GTX131276, lot number: 41,836), anti-Src monoclonal antibody (Cell signaling, catalog number: 2123, lot number: 5), anti-Ndrg1 polyclonal antibody (Proteintech, catalog number: 26902-1-AP, lot number: 00050845), anti-Hif1α polyclonal antibody (Proteintech, catalog number: 20960-1-AP, lot number: 00149455), anti-Stat3 polyclonal antibody (Santa Cruz, catalog number: sc-7179, lot number: A069), anti-Stat3 monoclonal antibody (Cell signaling, catalog number: 4904P, lot number: 7), anti-phospho-Stat3 (Tyr705) monoclonal antibody (Cell signaling, catalog number: 9145, lot number: 43), anti-β-actin monoclonal antibody (Santa Cruz, catalog number: sc-47778, lot number: l2822), and anti-mouse IgG(H + L), F(ab’)_2_ fragment (Cell signaling, Alexa Fluor 488 conjugate, catalog number: 4408, lot number: 24) were used in this study.

### Cell culture and generation of knockout cell lines

Huh-7.5 human hepatoma cells were kindly provided by Charles M. Rice (The Rockefeller University) and maintained in Dulbecco’s Modified Eagle Medium (DMEM, Fujifilm Wako Pure Chemical Corporation) supplemented with 10% fetal bovine serum (Sigma-Aldrich), 0.1 mM nonessential amino acids (Fujifilm Wako Pure Chemical Corporation), and 1% penicillin-streptomycin mixed solution (Nacalai Tesque, 09,367–34) at 37 °C in a humidified atmosphere containing 5% CO_2_ (7, 8). For HCV stock preparation and siRNA transfection, the medium was replaced with antibiotic-free medium.

To establish Src-KO cells, PX330-U6-Chimeric_BB-CBh-hSpCas9 (a gift from Feng Zhang; Addgene plasmid #42230; http://n2t.net/addgene:42,230; RRID: Addgene_42230), which encodes Cas9 and guide RNAs targeting either exon 5 or exon 10 of the human *Src* gene, was co-transfected into Huh-7.5 cells together with pcDNA3.1(−) (Invitrogen), which confers G418 resistance through the *neoR* gene. The following nucleotide sequences were used to design single-guide RNAs targeting Src exons. For exon 5: sense, 5′-TGTCCTTCAAGAAAGGCGAG-3′; antisense, 5′-CTCGCCTTTCTTGAAGGACA-3′. For exon 10: sense, 5′-GACCTGGAACGGTACCACCA-3′; antisense, 5′-TGGTGGTACCGTTCCAGGTC-3′. After G418 selection, resistant single-cell clones were isolated. Potential knockout clones were first screened by immunoblotting for Src protein expression and subsequently confirmed by direct sequencing of genomic DNA. Distinct insertion-deletion mutations (indels) were detected at the guide RNA target sites, resulting in frameshift mutations and the introduction of premature stop codons within the coding sequence. These findings confirmed the successful generation of Src-KO cell lines.

Abl-KO and Stat3-KO cell lines were generated as described in our previous reports (7, 32).

### Cell proliferation inhibition assay

For cell proliferation inhibition assays, approximately 1 × 10^5^ cells per well were seeded in 24-well plates and allowed to adhere overnight. The following day, cells were treated with four serially diluted concentrations of TKIs. After 72 h of treatment, live cell number was evaluated using the trypan blue exclusion method. To account for the 18-h half-life of Nilotinib, one-half of the original concentration was freshly added every 24 h to maintain effective drug levels. The optimal drug concentrations were determined based on proliferation outcomes. All experiments were performed in triplicate.

### Virus infection assays

HCVcc (44), which was derived from J6/JFH1 (45), was passaged in Huh-7.5 cells to produce viral stocks. Supernatants from infected cultures were collected for the determination of median tissue culture infectious doses (TCID_50_), as previously described (7). Serially diluted supernatants were inoculated into naïve Huh-7.5 cells in 96-well plates. Ninety-six hours post-infection, the cells were immunostained with anti-HCV core monoclonal antibody to identify core-positive cells, and TCID_50_ values were calculated accordingly.

For drug treatment assays, Huh-7.5 cells were seeded in 24-well plates 1 day prior to infection. Cells were infected with HCVcc at an MOI of 5 (TCID_50_/cell) in the presence of optimized concentrations of TKIs. After 24 h, the medium was replaced with fresh medium containing the same concentration of TKIs, and culture was continued for another 48 h.

For infection experiments in Src-KO cells, cells were seeded 1 day before infection and infected with HCVcc at an MOI of 5. Twenty-four hours post-infection, the medium was replaced, and the cells were cultured for an additional 48 h under standard conditions.

For HCV infection experiments in Ndrg1-knockdown cells, Huh-7.5 cells were transfected with siRNA targeting Ndrg1 (Ambion, Silencer^R^ Select Pre-designed siRNA, s20334, s20336; ThermoFisher Scientific siRNA negative control#1, lot number: ASO2L40 L) for 24 h, followed by infection with HCVcc at an MOI of 5. Six hours post-infection, the medium was refreshed and siRNA was re-administered. The cells were then maintained in culture for 72 h. For infection experiments in Ndrg1-overexpressing cells, cells were transfected with the pcDNA3.1-Ndrg1 plasmid for 6 h. The medium was then replaced with fresh complete medium, and cells were infected with HCV at an MOI of 5 overnight. The medium was replaced the next day, and the cells were maintained in culture for 72 h before subsequent analyses.

After each of the above treatments, supernatants were collected to measure extracellular HCV titers. To prepare intracellular HCV samples, infected cells were detached and suspended in distilled water, subjected to three cycles of freezing and thawing, and centrifuged at 2000*g* for 2 min. The supernatants were collected and the TCID_50_ values of both extracellular and intracellular samples were determined as described above.

### Immunostaining

Cells were washed once with PBS and fixed with cold methanol (−20 °C) for 20 min. After fixation, cells were blocked with normal goat serum (Vector Laboratories) for 30 min at room temperature. Cells were then incubated overnight at 4 °C with an anti-HCV core monoclonal antibody. After washing, Alexa Fluor 488-conjugated anti-mouse IgG (H + L), F(ab’)_2_ fragment, was used as the secondary antibody. Nuclei were counterstained with Hoechst 33258 (Dojindo Laboratories, Cellstain). Fluorescence images were acquired using an inverted fluorescence microscope (Olympus, Olympus IX71).

### Real-time quantitative PCR

Total RNA was extracted from cells using the High Pure RNA Isolation Kit (Roche). Reverse transcription was performed using ReverTra Ace qPCR RT Master Mix with gDNA Remover (Toyobo), with an appropriate amount of total RNA as input. Quantitative PCR was conducted using the SYBR FAST qPCR Kit (KAPA Biosystems) on a StepOne Plus Real-Time PCR System (Life Technologies). Primer sequences for HCV cDNA amplification were described previously (8). For Ndrg1, the following primers were used: forward, 5′-AGTCCTTCAACAGTTTGGGCT-3′; reverse, 5′-CATCCTGAGATCTTGGAGGCG-3′. Relative gene expression levels were calculated using the 2^−ΔΔCt^ method and normalized to the expression levels in the control group.

Extracellular HCV RNA was isolated from the supernatants of infected cell cultures using the QIAamp Viral RNA Mini Kit (QIAGEN). A standard curve was generated using viral RNA (9720 nucleotides) transcribed *in vitro* from pFL-J6/JFH1 (45), in which 1 μg of RNA corresponded to 1.936 × 10^11^ copies. The RNA was serially diluted to produce a range of standards. Viral RNA copy numbers were normalized to copies per microliter of supernatant and subjected to statistical analysis.

### Microarray analysis

Huh-7.5 cells were treated with Bosutinib as described in the virus infection assays section. Total RNA was extracted using the High Pure RNA Isolation Kit (Roche) according to the manufacturer’s instructions. RNA quality was evaluated using an Agilent 2100 Bioanalyzer (Agilent Technologies), and samples with RNA integrity number ≥ 9.0 were used for microarray analysis. Gene expression profiling was performed using the Clariom S Assay (ThermoFisher Scientific) and hybridized onto GeneChip arrays using the GeneChip System GCS3000. Raw CEL files were processed with the signal space transformation-robust multi-array average algorithm in Transcriptome Analysis Console software (ThermoFisher Scientific, v.4.0.3) for background correction, quantile normalization, and probe summarization. Differential expression analysis was conducted using the *limma* package (46) in *R* software (v.4.3.2). Genes with a |log_2_FC| > 0.5 and *p* < 0.05 were defined as DEGs. Visualization of DEGs, including volcano plots and hierarchical clustering, was performed in *R*. Gene Ontology enrichment analysis was performed in *R* using *clusterProfiler* (47) package. Adjusted *p*-values were calculated with the Benjamini-Hochberg method, and adjusted *p* < 0.05 was considered statistically significant.

### Generation of Ndrg1 overexpression plasmids and Hif1α knockdown experiments

To generate an Ndrg1 overexpression plasmids, the full-length Ndrg1 cDNA was reverse-transcribed from Huh-7.5 cells and subsequently amplified by PCR using specific primers (forward, 5′-GGGGGCTCGAGCTCGTCAGTTCACCATCCG-3′; reverse, 5′-GGGGAATTCACAGAGATCAGAGTCCGGGG-3′). The amplified product was digested with XhoI (Nippon gene) and EcoRI (Nippon gene), and subcloned into the pcDNA3.1(−) expression vector (Invitrogen). The recombinant plasmid was transformed into competent cells (DH5α) for amplification, and plasmid DNA was purified and verified by Sanger sequencing to confirm cDNA integrity. For transient transfection, cells were transfected with 1 μg of plasmid using Lipofectamine 3000 (Invitrogen) according to the manufacturer’s instructions. After 6 h, the culture medium was replaced with fresh complete medium, and the cells were maintained for 72 h. Protein lysates were then collected, and Ndrg1 overexpression was verified by immunoblotting.

For Hif1α knockdown experiments, Huh-7.5 cells were transfected with siRNAs targeting Hif1α (Ambion, Silencer^R^ Select Pre-designed siRNAs, s6539 and s6541) or a negative control siRNA (Thermo Fisher Scientific, siRNA Negative Control #1, lot: ASO2L40 L) for 96 h, after which protein lysates were prepared for immunoblot analysis.

### Immunoprecipitation and immunoblotting

For immunoprecipitation, cells were lysed in lysis buffer (50 mM Tris-HCl, pH 7.4; 150 mM NaCl; 10 mM EDTA; 100 mM NaF; 1 mM Na_3_VO_4_; 1% Triton X-100; 1 mM PMSF; and 2 μg/ml aprotinin), followed by incubation with a capture antibody or control IgG overnight at 4 °C. The lysates were then incubated with protein A/G agarose beads for 1 h at 4 °C on a rotating platform. Immunocomplexes were collected by centrifugation, washed four times with lysis buffer, and subsequently analyzed by SDS-PAGE.

For immunoblotting, detergent-soluble lysate samples were separated by SDS-PAGE gels and transferred onto polyvinylidene fluoride membranes (Merck Millipore). Membranes were blocked in Tris-buffered saline (25 mM Tris-HCl, pH 8.0, 150 mM NaCl) with 0.1% Tween-20 containing 5% nonfat dried milk for 1 h at room temperature, and then incubated with primary antibodies overnight at 4 °C. The following primary antibody dilutions were used: anti-HCV core (1:2000), anti-NS3 (1:2000), anti-Ndrg1 (1:8000), and anti-Src (1:2000), anti-Hif1α (1:3000), anti-Stat3 (1:2000), anti-phospho-Stat3 (1:2000). After washing, membranes were incubated with horseradish peroxidase-conjugated secondary antibodies (Jackson ImmunoResearch Laboratories) for 1 h at room temperature. Chemiluminescent signals were detected using Western Lightning ECL reagents (PerkinElmer Life Sciences) and visualized with LAS-3000 mini (Fujifilm).

To indicate molecular weight positions, Precision Plus Protein All Blue Standards (Bio-Rad, 1610373) were used. Band intensity was quantified using ImageJ software. To normalize protein expression levels, the median density of each target band was divided by that of the corresponding β-actin band.

### Statistical analysis

Results are presented as mean ± SD from at least three independent biological replicates. For normally distributed data with equal variances, two-tailed Student’s *t*-tests were used; for data with unequal variances, Welch’s *t*-tests were applied. For comparisons among more than two groups, one-way analysis of variance (ANOVA) was performed, followed by the Dunnett’s multiple comparisons test. Non-normally distributed data were analyzed using the Mann-Whitney *U* test. A *p* < 0.05 was considered statistically significant.

## Data availability

All data obtained in this study are included in the main figures and supporting information. Data obtained from the microarray analysis were deposited in the Gene Expression Omnibus (GEO) repository. To review GEO accession GSE305769: https://www.ncbi.nlm.nih.gov/geo/query/acc.cgi?acc=GSE305769 and enter token wlqbgowqdxibxix into the box.

## Supporting information

This article contains supporting information (7).

## Conflict of interest

The authors declare that they have no conflicts of interest with the content of this article.
