# Supplementary material for: Protein tyrosine kinase Src suppresses hepatitis C virus particle release through regulation of Ndrg1
Source: J Biol Chem. 2025 Dec 30;302(2):111125. doi: 10.1016/j.jbc.2025.111125 (PMC12860343; doi:10.1016/j.jbc.2025.111125)
Supplement: Supplementary Material [file mmc2.docx]

**Protein tyrosine kinase Src suppresses hepatitis C virus particle release through regulation of Ndrg1**

Leihua Fu^§¶^, Kenji Takeuchi^§‡^, Kazuyasu Chihara^§‡^, Weiying Feng^¶^, and Kiyonao Sada^§‡1^

^§^Department of Genome Science and Microbiology, Faculty of Medical Sciences, University of Fukui, Fukui 910-1193, Japan.

^‡^Organization for Life Science Advancement Programs, University of Fukui, Fukui 910-1193, Japan.

^¶^Department of Hematology, Shaoxing People’s Hospital, Shaoxing City, Zhejiang Province 312000, People’s Republic of China.

**Supporting Information List**

**Figure S1. Effects of TKIs on cell proliferation.**

**Figure S2. Quantification of extracellular HCV RNA copies in Huh-7.5 and Src-KO clones.**

**Figure S3. Abl-KO does not affect Ndrg1 expression.**

**Figure S4. Src-KO does not affect N-myc expression.**


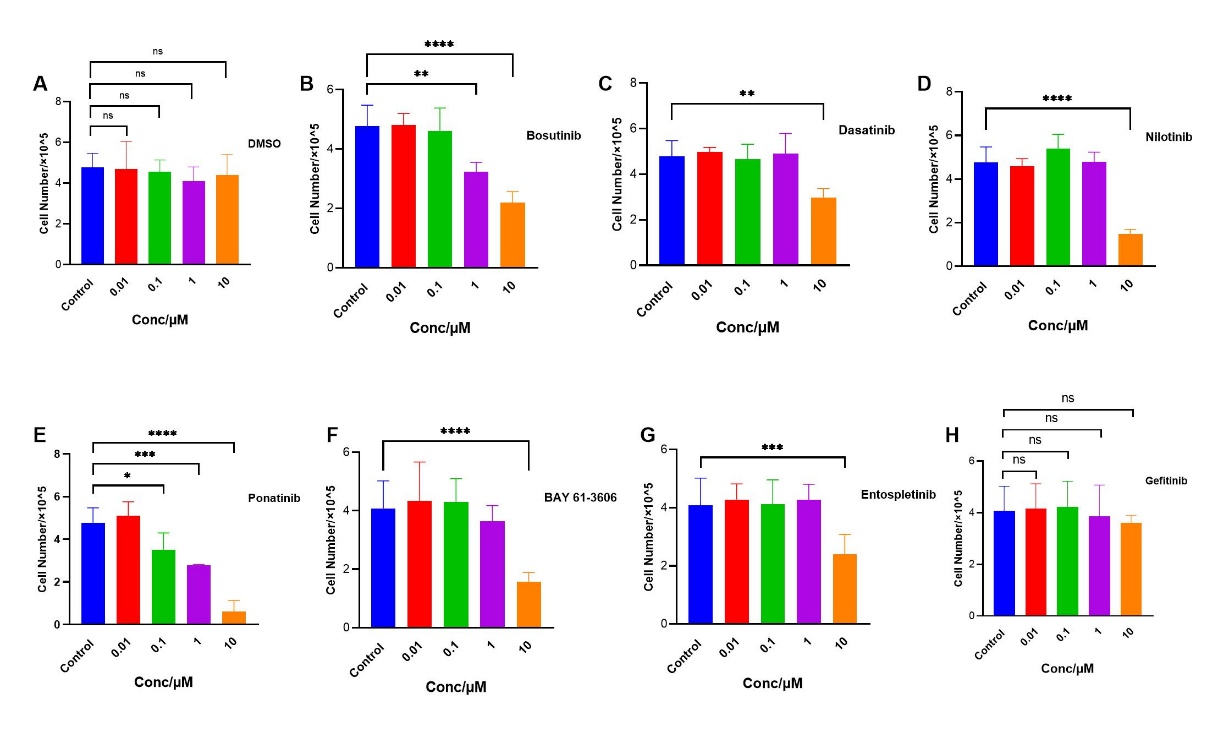


**Figure S1. Effects of TKIs on cell proliferation.** Cells were seeded in 24-well plates (1 × 10⁵ cells/well) and treated with serial dilutions (0.01, 0.1, 1, and 10 μM) of the indicated TKIs for 72 hours. Live cell numbers were quantified using the trypan blue exclusion method. For Nilotinib (*D*), due to its short half-life, half of the original concentration was replenished every 24 hours to maintain effective levels. Panels show the dose-dependent effects of (*A*) DMSO (vehicle control), (*B*) Bosutinib, (*C*) Dasatinib, (*D*) Nilotinib, (*E*) Ponatinib, (*F*) BAY 61-3606, (*G*) Entospletinib, and (*H*) Gefitinib. Data represent mean ± SD of three independent biological replicates. ns, not significant; **p* < 0.05; ***p* < 0.01; ****p* < 0.001; *****p* < 0.0001. (One-way ANOVA followed by the Dunnett’s multiple comparisons test).


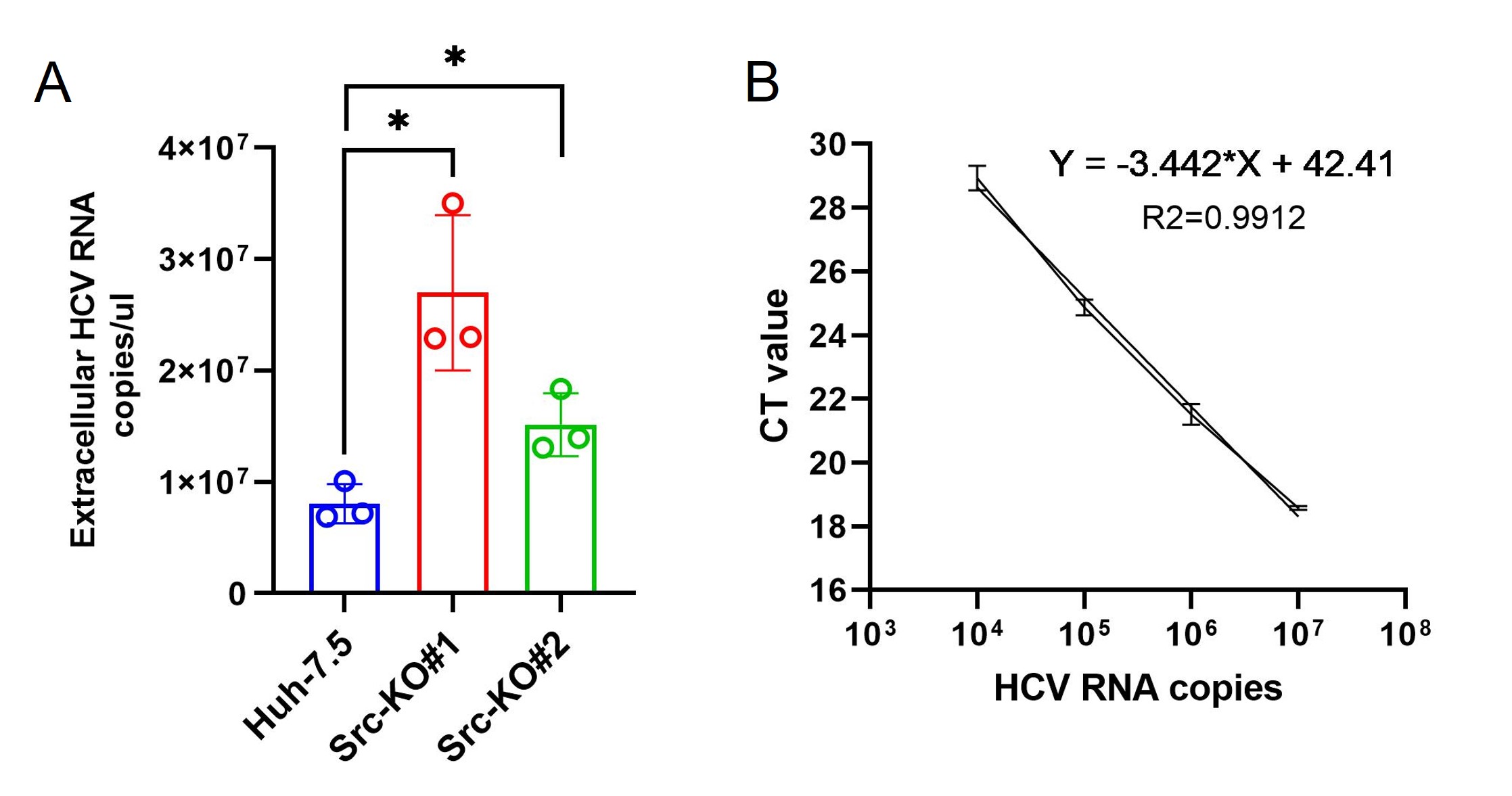


**Figure S2. Quantification of extracellular HCV RNA copies in Huh-7.5 and Src-KO clones.** *A*, Huh-7.5, Src-KO#1, and Src-KO#2 cells were infected with HCV as described in materials and methods and culture supernatants were collected at 72 hours post-infection. Extracellular HCV RNA was extracted using the QIAamp Viral RNA Mini Kit and quantified by RT-qPCR. Viral RNA copy numbers were calculated using a standard curve and normalized to copies per microliter of supernatant. Src-KO significantly increased extracellular viral RNA levels compared to wild-type cells. *B*, Standard curve generated by serial dilution of in vitro-transcribed HCV RNA (1 μg = 1.936 × 10¹¹ copies) showing linear correlation between input copy number and cycle threshold (CT) value (R² = 0.9912). Data represent mean ± SD of three independent biological replicates. Statistical significance was analyzed using One-way ANOVA followed by the Dunnett’s multiple comparisons test. **p* < 0.05.

**
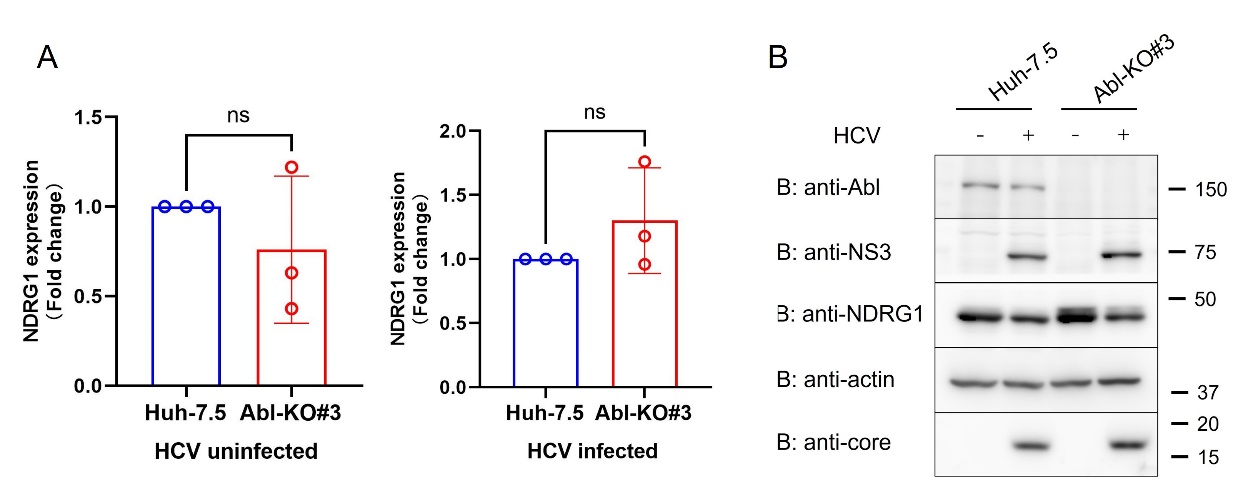
**

**Figure S3. Abl-KO does not affect Ndrg1 expression.** *A,* RT-qPCR analysis of Ndrg1 mRNA expression in Huh-7.5 and Abl-KO#3 clone (7) under uninfected (left) and HCV-infected (right) conditions. *B*, Immunoblot analysis confirmed that Ndrg1 protein expression remained unchanged in Abl-KO#3 cells compared to normal Huh-7.5 cells. Complete loss of Abl protein confirms successful knockout. Data represent mean ± SD of three independent biological replicates. Statistical significance was analyzed using Welch’s *t*-test. ns = not significant.


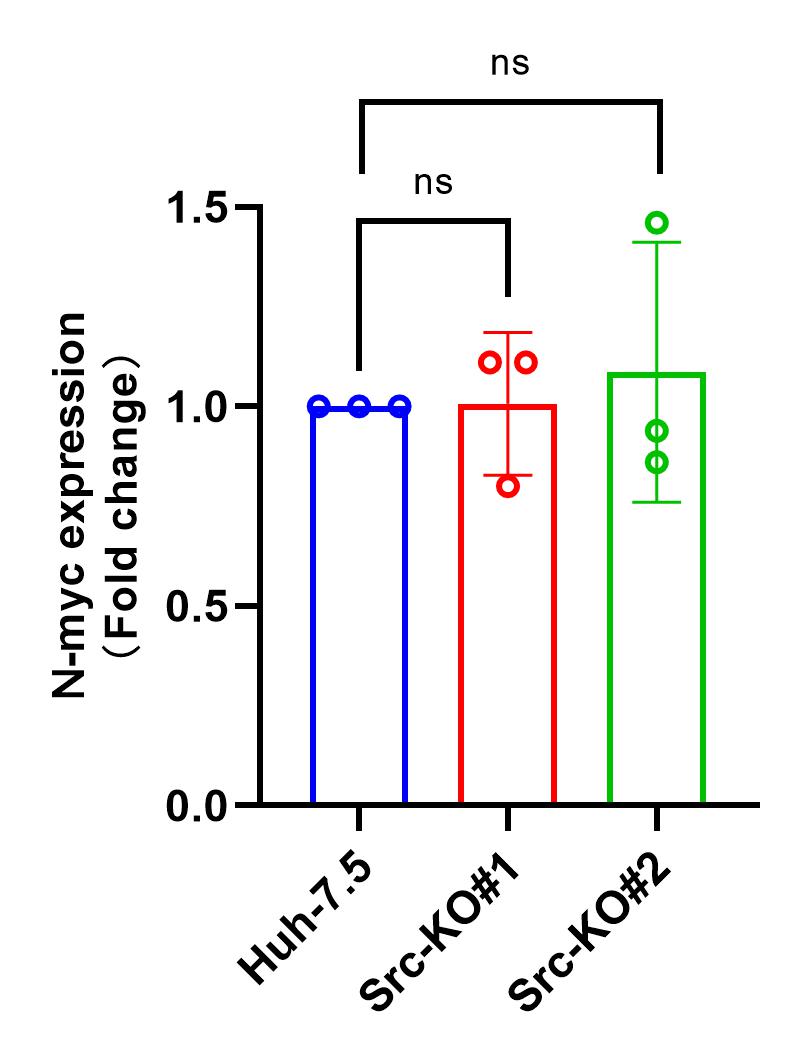


**Figure S4. Src-KO does not affect N-myc expression.** RT-qPCR analysis of N-myc mRNA expression in Huh-7.5 cells and two independent Src-KO clones. Data are presented as fold change relative to Huh-7.5 cells and represent mean ± SD of three independent biological replicates. Statistical significance was analyzed using Welch’s t-test. ns, not significant.

**Reference for Supporting Information**

7. Miyamoto, D., Takeuchi, K., Chihara, K., Fujieda, S., and Sada, K. (2022) Protein tyrosine kinase Abl promotes hepatitis C virus particle assembly via interaction with viral substrate activator NS5A *J Biol Chem* **298**, 101804
